# Clinical characteristics and assessment of risk factors in patients with influenza A-induced severe pneumonia after the prevalence of SARS-CoV-2

**DOI:** 10.1515/med-2024-0953

**Published:** 2024-04-15

**Authors:** Yujie Ma, Qiang Gao

**Affiliations:** Department of Cardiovascular Medicine, Dazhou Dachuan District People’s Hospital (Dazhou Third People’s Hospital), Dazhou, China; Department of Critical Care Medicine, Dazhou Central Hospital, No. 56 Nanyuemiao Street, Tongchuan District, Dazhou, 635000, Sichuan, China

**Keywords:** influenza A, severe pneumonia, coinfections, risk factors, mortality

## Abstract

**Purpose:**

The aim of this study is to describe the novel epidemiological and clinical characteristics of influenza A-induced severe pneumonia occurring after the prevalence of severe acute respiratory syndrome coronavirus 2 (SARS-CoV-2) and to further assess its potential risk factors for mortality.

**Methods:**

We retrospectively studied the consecutive case series of 30 patients with confirmed influenza A-induced severe pneumonia treated in the intensive care unit at Dazhou Central Hospital in Sichuan, China, from March 1 to April 30, 2023. Logistic regression was used to analyze the independent risk factors, and receiver operating characteristic (ROC) curves were applied to evaluate the predictive efficacy of associated risk factors for mortality.

**Results:**

The mortality rate was 33.3% in this study. Independent risk factors for mortality of patients were acute respiratory distress syndrome (ARDS) (*p* = 0.044) and septic shock (*p* = 0.012). ROC statistics for ARDS and septic shock to predict mortality in patients with influenza A-induced severe pneumonia demonstrated an area under the curve of 0.800 (sensitivity 80.0%, specificity 80.0%) and 0.825 (sensitivity 70.0%, specificity 95.0%), respectively.

**Conclusion:**

ARDS and septic shock were the independent risk factors for mortality in patients with influenza A-induced severe pneumonia following the end of the SARS-CoV-2 pandemic. But high level of next generation sequencing reads Aspergillus coinfection, and comorbidities did not increase death risk of the study population.

## 1 Introduction

With the end of the current coronavirus disease 2019 (COVID-19) pandemic in China, the epidemic of influenza A virus infection arrived suddenly and violently in March 2023, and influenza-associated hospitalization exceeded the peak levels of the past 3 years excessively [1]. Influenza virus infection is a worldwide health issue, sometimes progressing to severe pneumonia with substantial respiratory failure, acute respiratory distress syndrome (ARDS), multiorgan disfunction, and even death across all ages [2,3,4,5,6,7]. Existing evidence from China shows that approximately 4.5% of patients hospitalized with influenza A infection may develop severe pneumonia [8], and the in-hospital mortality of them is in the range of 1.2–1.9% [8,9,10].

Although the overall in-hospital mortality of patients with influenza A infection is relatively low, the in-hospital mortality rate due to influenza A-induced severe pneumonia remains to be as high as 20.6–41.6% [8,11,12,13]. Thus, the unacceptably high mortality rate of influenza A-induced severe pneumonia requires more research to deepen the understanding, optimize management, and guide clinical decision-making.

Previous studies from worldwide showed that cardiac disease, male gender, chronic pulmonary disease, immunosuppression, obesity, diabetes mellitus, and pregnancy were major risk factors associated with influenza severity [8,14]. However, the risk factors for mortality in patients with influenza A-induced severe pneumonia, especially after the high prevalence of severe acute respiratory syndrome coronavirus 2 (SARS-CoV-2), remains unknown. It is also unclear whether the influenza A-induced severe pneumonia widely spreading after the prevalence of SARS-CoV-2 manifests new characteristics. In this study, we systematically explore the emerging characteristics, and further appraise the outcomes and potential risk factors of influenza A-induced severe pneumonia following the end of the SARS-CoV-2 pandemic.

## 2 Materials and methods

### 2.1 Study design

We conducted a retrospective cohort study of adult patients with severe pneumonia who were admitted to a 60-bed comprehensive intensive care unit (ICU) between March 1, 2023 and April 30, 2023 at Dazhou Central Hospital in Sichuan, China. Patients (>18 years) with influenza A infection and who progressed severe pneumonia were included. The pathogens of severe pneumonia in our study were detected by metagenomic next generation sequencing (mNGS) [15,16]. Bronchoalveolar lavage fluid (BALF) samples were collected and sent to CapitalBio Medlab (Chengdu, China) for pathogen detection using mNGS analysis. We reviewed all medical records and collected the following data: demographics, clinical manifestations, comorbidities, laboratory findings on admission to ICU, complications, mNGS test results, and clinical outcomes.


**Ethical approval:** This study was approved by the Ethics Committee of Dazhou Central Hospital (No. 2024041).
**Informed consent:** Informed consent was not required because of the non-interventional and retrospective nature of the study.

### 2.2 Definitions

The diagnosis of severe pneumonia was consistent with the American Thoracic Society-Infectious Diseases Society of America guideline [17], including two major criteria and nine minor criteria. Major criteria included (1) invasive mechanical ventilation and (2) septic shock with the need for vasopressors. Minor criteria were (1) respiratory rate ≥30 breaths per min; (2) PaO2/FiO2 ≤250; (3) multi-lobar infiltrates; (4) confusion or disorientation; (5) blood urea nitrogen ≥ 20 mg/dL; (6) leucocyte count <4 × 10^9^ cells/L; (7) platelet count <100 × 10^9^ cells/L; (8) core temperature <36°C; and (9) hypotension requiring aggressive fluid resuscitation. The diagnosis of severe pneumonia required one of the major criteria or at least three minor criteria. In addition, ARDS was defined according to the Berlin definition [18]. Septic shock was defined if patients required a vasopressor to maintain mean arterial pressure ≥65 mmHg and serum lactate >2 mmol/L despite adequate fluid resuscitation [19]. 2.3 Statistical analysis

All statistical procedures were analyzed by the software package SPSS 22.0 for Windows (SPSS Inc., Chicago, IL, United States). Chi-square test or Fisher’s exact test was used for analyzing categorical variables. Continuous variables were expressed as mean value ± standard deviation (SD) and compared using Student’s *t*-test. Logistic regression was applied to analyze independent risk factors. The receiver operating characteristic (ROC) curve was used to further evaluate the predictive efficacy. *p* < 0.05 was considered statistically significant.

## 3 Results

### 3.1 Patients

During the two-month study period, 30 patients with confirmed influenza A-induced severe pneumonia were enrolled. All patients received conventional-dose (150 mg/day) oseltamivir for the therapy of influenza A. The baseline and clinical characteristics of patients are shown in Table 1. The mean age was 67 years (mean value ± SD, 67.23 ± 10.93; range, 46–85 years), and 17 (56.7%) patients were male. The average durations from first symptoms to ICU admission were 15 days (mean value ± SD, 15.30 ± 23.55; range, 3–120 days). The subtype of influenza A virus was H1N1 (23 [76.7%]). Another subtype of influenza A virus was H3N2 (7 [23.3%]). The most common pathogenic bacteria were gram positive bacteria (13 [43.3%]). The proportion of Aspergillus was 30% (9/30). It was worth noting that all patients had been infected with SARS-CoV-2 in the past 6 months. Of 30 severe cases, the mortality rate was 33.3% (10/30).

**Table 1:** Baseline and clinical characteristics of patients with influenza A-induced severe pneumonia

Parameters	Total (*n* = 30)	Survival (*n* = 20)	Death (*n* = 10)	*p*
Age, years	67.23 ± 10.93	65.20 ± 10.98	71.30 ± 10.12	0.15
Sex				0.80
Male	17 (56.7%)	11 (55.0%)	6 (60.0%)	
Female	13 (43.3%)	9 (45.0%)	4 (40.0%)	
Subtype of influenza A virus				0.37
H1N1	23 (76.7%)	14 (70.0%)	9 (90.0%)	
H3N2	7 (23.3%)	6 (30.0%)	1 (10.0%)	
NGS reads of influenza A virus (mean value ± SD)	14996.07 ± 20254.39	16015.95 ± 20617.70	12956.30 ± 20433.78	0.70
Other pathogens				
Gram positive bacteria	13 (43.3%)	8 (40.0%)	5 (50%)	0.71
Gram negative bacteria	10 (33.3%)	7 (35.0%)	3 (30.0%)	>0.99
*Candida*	10 (33.3%)	5 (25.0%)	5 (50.0%)	0.23
*Aspergillus*	9 (30.0%)	5 (25.0%)	4 (40.0%)	0.43
Onset of symptom to ICU admission, days	15.30 ± 23.55	7.70 ± 3.88	30.50 ± 37.02	0.08
APACHE II score	21.83 ± 6.08	19.85 ± 5.65	25.80 ± 5.03	0.009
SARS-CoV-2 infection^*^	30 (100.0%)	20 (100.0%)	10 (100.0%)	—
Length of IMV, h	135.03 ± 115.79	118.30 ± 109.14	168.50 ± 127.24	0.27
Length of hospital stay, days	12.53 ± 6.57	9.25 ± 5.50	7.90 ± 5.88	0.54
Symptoms				
Cough	28 (93.3%)	19 (95.0%)	9 (90.0%)	>0.99
Expectoration	25 (83.3%)	17 (85.0%)	8 (80.0%)	>0.99
Dyspnea	25 (83.3%)	15 (75.0%)	10 (100.0%)	0.14
Fever	12 (40.0%)	8 (40.0%)	4 (40.0%)	>0.99
Fatigue	5 (16.7%)	5 (25.0%)	0	0.14
Anorexia	4 (13.3%)	3 (15.0%)	1 (10.0%)	>0.99
Myalgia	2 (6.7%)	2 (10.0%)	0	0.54
Dizziness	1 (3.3%)	1 (5.0%)	0	>0.99
Vomiting	1 (3.3%)	1 (5.0%)	0	>0.99
Comorbidities				
COPD	15 (50.0%)	11 (55.0%)	4 (40.0%)	0.44
Hypertension	13 (43.3%)	10 (50.0%)	3 (30.0%)	0.44
Cardiovascular disease	8 (26.7%)	5 (25.0%)	3 (30.0%)	>0.99
Diabetes	6 (20.0%)	4 (20.0%)	2 (20.0%)	>0.99
Bronchiectasis	4 (13.3%)	4 (20.0%)	0	0.27
Malignancy	2 (6.7%)	2 (10.0%)	0	0.54
HIV infection	1 (3.3%)	1 (5.0%)	0	>0.99
Complications				
ARDS	12 (40.0%)	4 (20.0%)	8 (80.0%)	0.004
Septic shock	8 (26.7%)	1 (5.0%)	7 (70.0%)	<0.001
Acute liver failure	2 (6.7%)	0	2 (20.0%)	0.10
Acute renal failure	2 (6.7%)	0	2 (20.0%)	0.10

^*^SARS-CoV-2 infection in the past 6 months. Abbreviations: SD, standard deviation; NGS, next-generation sequencing; ICU, intensive care unit; APACHE II, Acute Physiology and Chronic Health Evaluation II; SARS-CoV-2, severe acute respiratory syndrome coronavirus 2; IMV, invasive mechanical ventilation; COPD, chronic obstructive pulmonary disease; ARDS, acute respiratory distress syndrome.

### 3.2 Symptoms and complications

The primary prodromal symptoms were cough (28 [93.3%]), expectoration (25 [83.3%]), dyspnea (25 [83.3%]), and fever (12 [40.0%]). Only 4 (13.3%) patients had no comorbidities. COPD (15 [50.0%]), hypertension (13 [43.3%]), cardiovascular disease (8 [26.7%]), and diabetes (6 [20.0%]) were the most common coexisting conditions. The main complications were ARDS (12 [40.0%]) and septic shock (8 [26.7%]).

### 3.3 Univariate analyses

The differences in baseline and clinical characteristics between survivors and non-survivors are demonstrated in Table 1. The univariate analysis showed that there were significant differences in APACHE II score (*p* = 0.009), ARDS (*p* = 0.004), and septic shock (*p* < 0.001) between the survival and death groups. As shown in Table 2, univariate analysis of laboratory findings on admission to ICU revealed that white blood cell count (*p* = 0.05) and lactate (*p* = 0.037) in the death group were significantly higher than that in the survival group.

**Table 2:** Laboratory findings of patients with influenza A-induced severe pneumonia on admission to ICU

Parameters	Mean value ± SD			*p*
	Total (*n* = 30)	Survival (*n* = 20)	Death (*n* = 10)	
PaO2:FiO2, mmHg	175.62 ± 69.81	192.72 ± 72.22	141.41 ± 52.37	0.06
White blood cell count, ×10^9^/L	12.92 ± 8.19	10.87 ± 7.56	17.03 ± 8.19	0.05
Neutrophil count, ×10^9^/L	11.28 ± 7.71	9.45 ± 7.24	14.95 ± 7.64	0.06
Lymphocyte count, ×10^9^/L	0.82 ± 0.81	0.71 ± 0.44	1.02 ± 1.27	0.47
Hemoglobin, g/L	117.87 ± 23.71	118.85 ± 23.52	115.90 ± 25.25	0.75
Platelet count, ×10^9^/L	177.90 ± 106.54	178.30 ± 107.76	177.10 ± 109.99	0.98
Prothrombin time, s	13.28 ± 3.37	12.53 ± 2.81	14.78 ± 4.02	0.08
APTT, s	29.86 ± 4.09	29.46 ± 3.88	30.67 ± 4.59	0.46
INR	1.16 ± 0.29	1.10 ± 0.24	1.29 ± 0.34	0.09
Fibrinogen, g/L	4.41 ± 1.65	4.63 ± 1.75	3.95 ± 1.39	0.29
D-dimer, ng/mL	2185.40 ± 3927.69	1881.65 ± 4120.77	2792.90 ± 3639.39	0.56
Total bilirubin, µmol/L	17.36 ± 27.18	10.16 ± 7.00	31.75 ± 43.95	0.16
Direct bilirubin, µmol/L	9.10 ± 15.50	5.15 ± 4.45	17.01 ± 25.05	0.17
Alanine aminotransferase, U/L	156.70 ± 355.64	97.95 ± 240.38	274.20 ± 512.40	0.32
Aspartate aminotransferase, U/L	434.60 ± 1442.43	178.25 ± 546.13	947.30 ± 2374.15	0.34
Blood urea nitrogen, mmol/L	8.70 ± 4.21	7.73 ± 3.89	10.64 ± 4.33	0.07
Creatinine, µmol/L	75.69 ± 29.76	73.15 ± 30.34	80.78 ± 29.45	0.52
Albumin, g/L	32.64 ± 4.73	32.60 ± 4.88	32.72 ± 4.65	0.95
Lactate, mmol/L	3.04 ± 1.94	2.37 ± 1.12	4.38 ± 2.54	0.037
C-reactive protein, mg/L	77.80 ± 45.75	78.32 ± 46.28	76.77 ± 47.11	0.93
Procalcitonin, ug/L	3.27 ± 8.19	3.90 ± 9.68	2.00 ± 3.94	0.56

Abbreviations: ICU, intensive care unit; SD, standard deviation; PaO2, partial pressure of oxygen; FiO2, fraction of inspired oxygen; APTT, activated partial thromboplastin time; INR, international normalized ration.

### 3.4 Logistic regression analysis and ROC analysis

Significant variables in the univariate analysis were included in the logistic regression analysis performed using the forward selection approach. The other variables with p value less than 0.50 were also included in the logistic regression analysis in order to avoid missing important risk factors. As shown in Table 3, ARDS (*p* = 0.044) and septic shock (*p* = 0.012) were independent risk factors for mortality of patients with influenza A-induced severe pneumonia. And further ROC statistics for ARDS and septic shock to predict mortality showed an area under the curve (AUC) of 0.800 (sensitivity 80.0%, specificity 80.0%) and 0.825 (sensitivity 70.0%, specificity 95.0%), respectively (Table 4).

**Table 3:** Multivariate logistic regression analysis for mortality

Items	B	SE	Wald	df	OR	95% CI	*p*
ARDS	2.533	1.258	4.056	1	12.589	1.070–148.073	0.044
Septic shock	3.576	1.422	6.319	1	35.720	2.198–580.415	0.012
Constant	−3.033	1.086	7.804	1	0.048	—	0.005

Abbreviations: SE, standard error; OR, odds ratio; CI, confidence interval; ARDS, acute respiratory distress syndrome.

**Table 4:** Results of ROC curve analysis

Items	AUC	SE	*p*	95% CI	Sensitivity	Specificity
ARDS	0.800	0.091	0.008	0.622–0.978	0.800	0.800
Septic shock	0.825	0.094	0.004	0.641–1.000	0.700	0.950

Abbreviations: ROC, receiver operating characteristic; AUC, area under the curve; SE, standard error; CI, confidence interval; ARDS, acute respiratory distress syndrome.

## 4 Discussion

In this study, all patients had been infected with SARS-CoV-2 in the past 6 months. So, we describe the clinical features of influenza A-induced severe pneumonia occurring after the prevalence of SARS-CoV-2. Influenza A virus infection is usually a self-limited disease, but some patients need to be hospitalized or even admitted to the ICU. As we mentioned above, 4.5% of patients hospitalized with influenza A infection may develop severe pneumonia, and the mortality of inpatients with influenza A-associated severe pneumonia during the 2017–2018 influenza season in Zhejiang, China was up to 41.6% [8]. However, in our study, the 33.3% in-hospital mortality of influenza A-induced severe pneumonia after the prevalence of SARS-CoV-2 was lower than the 41.6% in-hospital mortality reported by Zou et al. [8]. All patients in our study received conventional-dose (150 mg/day) oseltamivir. Of 122 oseltamivir-administered patients, in supplementary material provided by Zou et al. [8], 64 (52.5%) received single dose oseltamivir (150 mg/day) and had a higher 60-day mortality rate (50.0%).

We subtyped the influenza A virus by next generation sequencing (NGS). Of 30 cases with influenza A-induced severe pneumonia in our study, H1N1 (76.7%) was predominant during the study period. The trend of prevalence was consistent with the national surveillance reports [1]. Pandemic H1N1 emerged in Mexico in March 2009 [20]. The first reports of human infection with H3N2 were in 1968 [21]. The mechanisms of influenza infections enhancing disease severity involve host, viral, and bacterial factors [22]. Among them, virus factors may include virus type/subtype [8]. However, in our results, there was no statistical difference in the proportion of virus subtypes between the survival group and death group. Subtype of influenza A virus was not the risk factor for mortality in patients with influenza A-induced severe pneumonia. In addition, a study in mice recently has shown that coinfection with influenza A (H1N1) virus and SARS-CoV-2 increases disease severity and impairs neutralizing antibody and CD4^+^ T cell responses [23].

Bacterial pneumonia following an influenza A virus infection is one of the major causes of morbidity and mortality. During the 1918 influenza pandemic, the death of infected population was associated with bacterial infections, primarily with Gram positive bacteria [24]. Our results also suggested that Gram positive bacteria (43.3%) were the main pathogens besides influenza A virus. During the 2009 H1N1 pandemic, bacterial infections account for 34% of H1N1 infections admitted to the ICU around the world [25]. Histologic and microbiologic autopsy evidence of bacterial pneumonia was detected in 55% of patients who died following confirmed H1N1 infection [26]. Influenza A can inhibit Th17 immunity and increase susceptibility to secondary bacterial pneumonia [27]. Furthermore, the endoplasmic reticulum chaperone glycoprotein 96 (GP96) drives exacerbation of secondary bacterial pneumonia following influenza A virus infection [28].

In our study, we revealed a high incidence rate of Aspergillus coinfection (30.0%) in patients with influenza A-induced severe pneumonia. The mortality of influenza A-induced severe pneumonia coinfected with Aspergillus was 44.4% (4/9). Regrettably, few studies have reported the coinfection of Aspergillus in patients with influenza A-induced severe pneumonia. Beumer et al found that Aspergillus fumigatus was the most common fungal pathogen 8/45 (17.8%) in ICU patients with influenza-associated pneumonia [29]. Ku et al. reported that the in-ICU mortality of severe influenza patients with invasive pulmonary aspergillosis was up to 66.7% (14/21) [30].

Different responses of the host to influenza virus infection also affect disease severity. Male gender, chronic pulmonary disease, and diabetes mellitus are the independent risk factors for influenza A-associated pneumonia severity [8]. Cardiac disease, immunosuppression, obesity, and pregnancy are associated with influenza severity [14]. Severe and fatal influenza virus infections are related to hemorrhagic bronchitis, bronchiolitis, and alveolitis with pulmonary edema and hemorrhage [31]. Our results suggested that ARDS and septic shock were independent risk factors for mortality of patients with influenza A-induced severe pneumonia. But the difference in pathogens, comorbidities, and even the symptoms is not statistically significant between the survival group and death group.

Additionally, it should be noted that although more reads of NGS means higher sensitivity to a great extent [15], NGS reads was not the risk factor associated with mortality in patients with influenza A-induced severe pneumonia in this study. Of course, our study has several limitations. First, this is a retrospective study with a relatively small sample size, causing possible risk factors to be missed. And the nature of retrospective research cannot confirm the causal relationship between independent risk factors and death. Second, NGS technique itself has certain limitations. Due to the influence of original microbial concentration in BALF and detection range, NGS cannot detect all pathogens. Third, we just investigated the laboratory findings of patients on the first day of admission to the ICU. It could not reflect the dynamic progression of the disease. Fourth, we cannot evaluate the morbidity of influenza A-induced severe pneumonia due to the deficiency of data on total influenza A patients outside the ICU during the study period.

## 5 Conclusion

The present study demonstrated that higher APACHE II score, white blood cell counts, and lactate, and more frequency of ARDS and septic shock were observed in dead patients with influenza A-induced severe pneumonia. ARDS and septic shock were independent risk factors for mortality of patients with influenza A-induced severe pneumonia following the end of the SARS-CoV-2 pandemic and showed good predictive efficacy for mortality. In addition, NGS reads, Aspergillus coinfection, and comorbidities were not the risk factor associated with mortality in our study. Further studies with larger sample sizes are needed to evaluate the risk factors model in patients with influenza A-induced severe pneumonia.
